# The Challenges in Designing a Prevention Chatbot for Eating Disorders: Observational Study

**DOI:** 10.2196/28003

**Published:** 2022-01-19

**Authors:** William W Chan, Ellen E Fitzsimmons-Craft, Arielle C Smith, Marie-Laure Firebaugh, Lauren A Fowler, Bianca DePietro, Naira Topooco, Denise E Wilfley, C Barr Taylor, Nicholas C Jacobson

**Affiliations:** 1 Department of Psychiatry and Behavioral Sciences Stanford University School of Medicine Palo Alto, CA United States; 2 Center for m2Health Palo Alto University Los Altos, CA United States; 3 Department of Psychiatry Washington University School of Medicine St Louis, MO United States; 4 Department of Behavioural Sciences and Learning Linköping University Linköping Sweden; 5 Center for Technology and Behavioral Health Geisel School of Medicine Dartmouth College Lebanon, NH United States

**Keywords:** chatbot, eating disorders, digital mental health, prevention, intervention development

## Abstract

**Background:**

Chatbots have the potential to provide cost-effective mental health prevention programs at scale and increase interactivity, ease of use, and accessibility of intervention programs.

**Objective:**

The development of chatbot prevention for eating disorders (EDs) is still in its infancy. Our aim is to present examples of and solutions to challenges in designing and refining a rule-based prevention chatbot program for EDs, targeted at adult women at risk for developing an ED.

**Methods:**

Participants were 2409 individuals who at least began to use an EDs prevention chatbot in response to social media advertising. Over 6 months, the research team reviewed up to 52,129 comments from these users to identify inappropriate responses that negatively impacted users’ experience and technical glitches. Problems identified by reviewers were then presented to the entire research team, who then generated possible solutions and implemented new responses.

**Results:**

The most common problem with the chatbot was a general limitation in understanding and responding appropriately to unanticipated user responses. We developed several workarounds to limit these problems while retaining some interactivity.

**Conclusions:**

Rule-based chatbots have the potential to reach large populations at low cost but are limited in understanding and responding appropriately to unanticipated user responses. They can be most effective in providing information and simple conversations. Workarounds can reduce *conversation errors*.

## Introduction

### Eating Disorders Prevention

Eating disorders (EDs) are serious psychiatric disorders associated with high morbidity and mortality, marked psychosocial impairment, and poor quality of life [1]. A recent meta-analysis found EDs prevalence rates of about 8% in women in the Western world, as well as evidence that these disorders are prevalent worldwide [2]. However, fewer than 20% of individuals who develop EDs receive treatment [3,4]. EDs prevention plays a vital role to help bridge the treatment gap. Fortunately, a number of risk factors for EDs onset have been identified, including internalization of the thin-body ideal and having many weight and shape concerns [5-7]. Weight and shape concerns and internalization of the thin-body ideal affect many young women. In one study, about 23% of college-age women had elevated levels of weight and shape concern that put them at risk for EDs [3]. Therefore, prevention of EDs is of utmost importance given the prevalence and low treatment rates of affected individuals. Interventions designed to target the highest risk groups have been shown to reduce risk factors and even onset [7-9]. For instance, a recent meta-analysis found a 38% decrease in incidence in the intervention groups compared with controls with small to moderate effects on EDs symptoms and risk factors, with most of the evidence coming from internet-based studies [8].

On the assumption that internet-based programs can provide easy and convenient access to EDs prevention, we developed a cognitive behavior–based program called Student Bodies (C). Human-moderated (guided) versions of Student Bodies have been shown to be associated with moderate improvements in ED-related attitudes, including reductions in negative body image and the desire to be thin [9-12]. Human moderators helped reinforce the use of the program by providing support and feedback. When comparing a moderated and an unmoderated version of the Student Bodies program, it was found that some guidance and encouragement from a human moderator improved outcomes [13]. However, providing human moderation to prevention programs incurs cost and is not a viable approach to reaching large populations who might benefit from EDs risk reduction.

### Chatbot Development

As such, we considered ways to provide *automated moderation* and specifically considered whether a chatbot could provide some automated interactivity, mirroring 1 aspect of human moderation. Chatbots are computer programs that can provide information and simulate human conversations [14]. Chatbots are widely used in the United States for several activities (eg, Siri, Alexa, or service centers) [15]. In recent years, many chatbots have been developed to provide psychoeducational and mental health interventions [16]. Chatbots also have the advantage of being delivered via mobile devices. As of 2019, 96% of American adults aged between 18 and 29 years owned a smartphone [17], smartphone users have an average screen time of 3 hours 10 minutes per day [18], and millennials spend nearly 50 minutes each day texting [19]. Research has found that, relative to an internet browser–based program, a chatbot-based program was associated with higher ease of use and increased response rate [20,21]. While chatbots in various forms are becoming widespread, few studies have evaluated their effectiveness in the prevention of mental health problems [14,22,23].

Chatbots can be developed in several ways [24-26]. One approach is to write out the basic conversations, including responses to user inputs, and then continue to refine the conversations based on user and chatbot inputs. In other words, it is necessary to develop a hand-curated, rule-based chatbot. An advantage of this is that the responses can be prescripted and controlled by the investigators. A disadvantage is that the conversations are predefined and thus limited. Another basic approach is to use artificial intelligence to generate responses in which the chatbot learns responses based on exemplar data [27]. Exemplar data for generative chatbots can be formed through prior chatbot interactions and can be curated through both user and expert ratings [28]. Generative chatbots work by mimicking the semantic patterns of the pre-established narrative text on which it is trained. An advantage is that conversations can be dynamic and fluid, adopting a wide repertoire, but it requires large, curated databases as well as considerable technical expertise. In addition, many chatbots have retrieval-based algorithms running to identify potential user inputs with authored text (ie, sometimes called intent-matching). In mental health chatbots, they can be used in areas outside the scope of the core content, such as suicidality [29,30].

Unfortunately, most researchers and providers who might consider the benefit of developing a chatbot do not have the resources to develop an artificial intelligence–based chatbot. However, rule-based chatbot authoring programs are becoming available and can be used without extensive programming experience (eg, Rasa [31] and Google’s DialogFlow [32]). Such an approach also represents a reasonable first-line approach to the initial development of a chatbot for a specific purpose. Therefore, we decided to use this approach to create a chatbot for Student Bodies in an effort to develop a scalable and low-cost resource for those in the United States who might benefit from Student Bodies. Our goal is to create an automated version of the program called the Body Positive program. Body Positive is moderated by a chatbot called Tessa (TM), developed by a private mental health chatbot company, X2AI.

The development of EDs prevention chatbots is in its infancy. A systematic review in 2019 found that only 1 out of 41 mental health chatbots targeted EDs [33]. Since the publication of the 2019 systematic review, Beilharz et al [34] published a paper on the acceptability and feasibility of a chatbot that provides psychoeducation and coping skills targeting people with body image and eating concerns. Despite increasing attention on the use of chatbots for mental health treatment and prevention, there is relatively little information in the literature about the process of developing and refining mental health–related rule-based chatbots. This could be of great value for those designing such chatbots in the future. Therefore, the objective of this study is to share our examples of challenges and workarounds in designing and refining a rule-based EDs prevention chatbot that targets young adult women at risk of developing an ED, to be evaluated in a separate randomized trial.

## Methods

### Body Positive

Body Positive is an EDs prevention program delivered by a chatbot, Tessa, that targets women between the ages of 18 and 30 years who are at risk for developing an ED. Body Positive was designed to be tested in a randomized controlled trial, the results of which were published in a separate paper [35]. The procedures and materials used in this study were approved by the institutional review board of Palo Alto University. All procedures performed in this study were in accordance with the ethical standards of the 1964 Declaration of Helsinki and its later amendments or comparable ethical standards.

Body Positive was modified from the original Student Bodies [10,36] prevention program. The final version of Body Positive consisted of an introduction and 8 sessions. The introduction covered information about the program, privacy, protocol for crisis, and the limitations of the chatbot (eg, not moderated by a person in real time and will say things that may seem off). The 8 conversations covered the core content of the original Student Bodies program, which included content that addressed challenging the thin-body ideal, media literacy, 4Cs (comparisons, conversations, commercials, and clothing), healthy eating, critical comments, exercise, bingeing, and maintenance [13]. One common strategy for developing chatbots is to use a rule-based approach in which investigators create and modify the scripts and algorithms that drive the chatbot’s conversation [37]. This is the approach we followed. These conversations were programmed into a chatbot, and the chatbot initiated each conversation in a predetermined order. Participants were encouraged to complete 2 conversations a week.

The chatbot that delivered and moderated Body Positive was fully automated. In addition to the Body Positive-specific modules, there were other pre-existing modules (ie, a crisis module and a module that was deployed if cursing was detected) and functions (ie, opting out of program reminders and recognizing and responding to questions) available from the wider X2AI chatbot platform that were triggered based on keywords (eg, “Unsubscribe” or “?”) in users’ comments. Chatbot conversational dynamics were meant to mimic natural text-based conversations. For example, the chatbot sent a message, or sometimes a few messages in succession, and then the users would respond, as instructed in the introduction, to continue the conversation. The communication was synchronous, as the chatbot was designed to respond to each of the user comments within seconds.

### Guiding Principles for Chatbot-Specific Content Development

There were several general principles that guided the process of modifying the Student Bodies content for the interactive chatbot platform.

**Figure 1:**
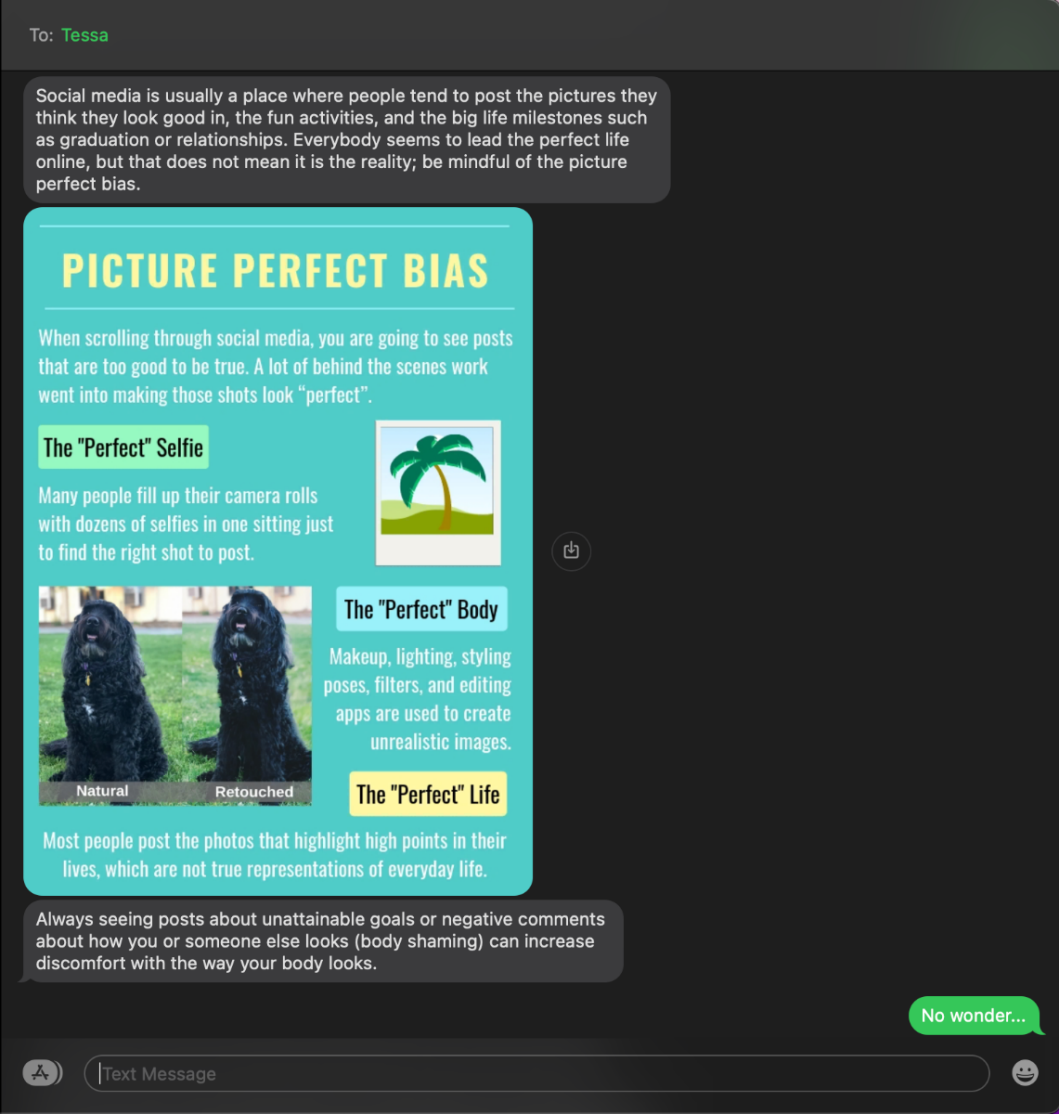
A screenshot of an infographic embedded as part of the text.

First, the length of each chatbot response was kept short to align with the texting culture. The standard SMS text message character limit was 160 characters. Therefore, each chatbot response was ideally fewer than 80 characters for short responses or 160 characters for longer sentences. To improve readability, only a maximum of 3 short responses or 1 long response would be sent in a row to keep new content visible on most phone screens without scrolling. A challenge was to provide an adequate discussion of the topics at hand within such word limitations. To help reinforce ideas and to break up *walls* of text, we created a series of infographics (Figure 1) covering material discussed in the sessions that were incorporated in the conversations. Infographics were embedded as part of the SMS text messages where users could view immediately without clicking any links. We encouraged users to take screenshots for later review.

Second, the chatbot responses were designed to convey support and warmth and to be appropriate for most users. We achieved this by providing reinforcement, encouragement, and supportive language. The conversations were designed to be interactive, such that a user could respond to an open-ended question with a sentence. The chatbot also used emojis, in addition to infographics, with the goals of making the program more interesting and aligning with current texting culture or standards [38]. The chatbot had a proprietary artificial intelligence algorithm that detected the valence (positive, negative, and unsure) of user responses, which allowed us to deliver the nuanced responses of warmth and support appropriately.

Our first priority was to author a rule-based, interactive chatbot (as opposed to a program driven by artificial intelligence), focusing on creating interactivity. We anticipated that we would need to continue to improve the conversations over time, following the process described in subsequent sections. Once this pilot program is evaluated and widely deployed, if proven effective, we would be able to generate more transcript exemplar data. It would then be possible to further improve the core program by using machine learning and related tools.

### Participants

Participants were recruited through web-based social media platforms, such as Facebook and Instagram advertisements, working with Instagram influencers, physical flyers posted on university campuses and other public community settings, and referrals through the National Eating Disorders Association web-based screen [39] or other ongoing EDs research studies.

A total of 210 participants completed a web-based screening survey [40]. The screen sought informed consent, baseline data, and eligibility. The inclusion criteria included being aged between 18 and 30 years, identifying as female, and screening as at risk for an ED. Participants were excluded if they did not meet the age or gender criteria, were not at risk for an ED, or screened positive for a clinical or subclinical ED. Participants who screened positive for an ED were provided with referral information, including information on how to access the National Eating Disorders Association web-based treatment provider database and helpline. When participants reached out to the chatbot via either Facebook Messenger or SMS text messaging, the chatbot asked for their user ID and then initiated the introduction conversation.

In addition, 2199 participants accessed the chatbot via either Facebook Messenger or SMS text messaging through social media recruitment. Users did not have to pay to access the chatbot.

### Measures

#### Eating Disorder Risk

EDs risk was determined using the Weight Concerns Scale [41], a 5-item self-report questionnaire that assesses weight and shape concerns, which has been shown to be a robust indicator of EDs risk [42]. There are three 5-point questions, one 4-point question, and one 7-point question that are transformed to yield a total score range of 0 to 100. High risk was defined as a score of 47 or above on the Weight Concerns Scale, indicating high weight and shape concerns. EDs risk was measured at all time points.

#### Eating Disorder Screen

ED diagnosis, an exclusion criterion, was measured using the Stanford-Washington University Eating Disorder (SWED) screen [43]. The SWED screen has been shown to have acceptable sensitivity (from 0.90 for anorexia nervosa to 0.55 for purging disorder) and specificity (from 0.99 for anorexia nervosa to 0.78 for subthreshold binge ED) for identifying an ED diagnosis [43,44]. ED diagnosis was measured using SWED screen at baseline only.

#### Transcript Review

The research team monitored the chatbot’s performance by reviewing the transcripts between the chatbot and users at least once a month. Over 150,000 responses (105,000 from the chatbot and 52,129 from users) were reviewed to identify bugs, chatbot responses that were erroneous or problematic, and conversations that did not flow smoothly. Transcripts were reviewed and evenly divided among 7 authors, who were mental health professionals and supervised and trained graduate and undergraduate students in psychology. Transcripts were reviewed monthly between December 2019 and May 2020. Each reviewer identified possible bugs that needed to be fixed and chatbot responses that needed to be improved. We did not create a *threshold* response or rubric but relied on reviewers’ judgment that the conversation should be reviewed by the group. Reviewers would present their transcript review findings to the group, focusing on issues that would negatively affect user experience. These issues and possible solutions were then discussed in weekly team meetings. Discussions and changes were monitored and tracked to ensure that they had been implemented. The chatbot content authoring platform was straightforward and accessible, which allowed the research team to easily and independently (ie, without the assistance of the technology partner) make iterative changes to the content and create fixes for minor bugs. In addition, the technology partner was responsive to troubleshooting technical glitches. The research team met with representatives from the company regularly for consultation and to implement major modifications and bug fixes.

#### Time Costs of Transcript Review

In general, each reviewer was given about 4000 to 5000 lines of transcript (including chatbot informational messages and user comments) each month, which required ≤2 hours to review. The total time devoted to transcript review was ≤60 hours over 6 months. In addition, eight 1-hour research meetings were held to review the transcripts and brainstorm solutions. Additional time was also required to implement these changes.

Consistent with Mohr et al [45], our general approach was not to change any of the core content or activities of the program but to improve the users’ experience by fixing bugs and reducing problematic chatbot responses through transcript review. Thus, the following section aims to address the problems and challenges we faced, namely, how to provide appropriate reinforcement; how to respond to users’ questions; and challenges with comprehensibility, context awareness, and technical issues.

## Results

### Overview

Between September 7, 2019, and May 31, 2020, we received 52,129 user comments from 2409 unique users who responded to the chatbot with at least one message. Participants were recruited from multiple sources. In this study, some interacted with the chatbot anonymously. As a result, demographic data for the entire sample were not reported. From March 10, 2020, onward, after many changes were made, we reviewed 26,305 lines of user comments and found only several minor errors that did not negatively impact users’ experience.

### How to Provide Reinforcement

#### Challenge

Authoring appropriate responses to nearly all user comments is one of the biggest challenges in creating a chatbot. For instance, our initial goal in creating the chatbot was to provide encouragement to continue with the program through positive responses, for example, “Great!” and “Wonderful!” While the positive responses were appropriate for many user responses, these positive responses did not work for some interactions. For example, when the chatbot asked, “Do you want to commit to NO FAT TALK, say for the next month?” The user replied, “Haha.” The prescripted response was “Wonderful! You might want to let your friends know that you are committed to NO FAT TALK for the next month.” We also found that positive responses unexpectedly reinforced harmful behaviors at times. For example, the chatbot prompted, “Please share with me a few things that make you feel good about yourself. For example, your humor, grace, personality, family, friends, achievements and more!” The user replied, “I hate my appearance, my personality sucks, my family does not like me, and I don’t have any friends or achievements.” The chatbot responded by saying, “Keep on recognizing your great qualities! Now, let’s look deeper into body image beliefs.” See Table 1 for additional examples.

**Table 1:** Additional examples of inappropriate chatbot reinforcement responses.

Issues	Examples	Solutions
Reinforcing potentially harmful behaviors	Chatbot: Now, please take a moment to write about when you felt best about your body? User 1: I have never felt good about myself. User 2: When I was underweight and could see my bones User 3: When I was skinnier I felt better because I could do more. I felt really good about my body when I went to the gym 5-6 days a week. User 4: I feel best about my body when I ignore it and don’t think about it at all Chatbot: It is awesome that you can recognize a moment when you felt confident in your skin, let’s keep working on making you feel this good more often.	Use of AI^a^ valence detection to deliver nuanced responses that match the tone of users’ comments (eg, positive, unsure, or negative) Replacing nonspecific positive responses with neutral statements
Inappropriate standardized positive response	Chatbot: This next exercise will help you learn to appreciate the many other aspects of yourself. Take a minute to write down a few things that make you feel good about yourself. For example, your humor, grace, personality, family, friends, achievements and more! User 1: I don’t have anything User 2: Ummmmm I actually kinda hate myself so we’re not going to get very far there. I think I’m a selfish piece of crap. User 3: I can’t think of anything. Chatbot: Keep on recognizing your great qualities! Now, let’s look deeper into body image beliefs.	Use of AI valence detection to deliver nuanced responses that match the tone of users’ comments (eg, positive, unsure, or negative) Replacing nonspecific positive responses with neutral statements

^a^AI: artificial intelligence.

#### Solution

To avoid reinforcing harmful comments, nonspecific and positive responses, for example, “Great!” and “Wonderful!” were mostly removed and replaced with more neutral statements while maintaining a warm tone. For example, the chatbot asked, “Share with me what you can say to yourself to minimize harmful comparisons.” The user replied, “Try to focus on the good things you have in common with others.” The chatbot responded, “Okay, keep challenging your thoughts when you compare yourself to others unrealistically.”

In addition to rewriting 1 standard positive chatbot response for the prompt that asked users to think of positive qualities about themselves, we added more nuanced responses to capture potential negative or ambivalent user comments. For the abovementioned example (eg, “I hate myself...”), the statement, “Sometimes it is difficult to remember the good qualities that you possess. You might consider something positive that someone who knows you well would say about you,” was added as a response to encourage and validate users who may be struggling with the prompt and to help users better engage with the content.

The following example highlights the complexity of crafting a positive response that “always works.” The chatbot asked, “What is a small healthy eating habit goal you would like to set up before you start your next conversation?” One user replied, “Don’t eat.” The chatbot said, “Take a moment to pat yourself on the back for doing this hard work, <<USER>>!” The chatbot was later updated to, “Thanks for taking the time to think about this, <<USER>>!” The updated language was specific to reinforcing the effort put into engaging in the exercise while not directly reinforcing the problematic response, that is, potential food restriction. However, the problematic response, that is, not eating, was not addressed. With many more responses, it would be possible to train the AI to identify and respond better to problematic responses.

#### Lesson Learned

It is difficult to write prescripted responses that are appropriate to all the varied comments that users make in response to chatbot questions. Praise words designed to respond to user activities may inadvertently reinforce harmful behaviors.

### How to Respond to Users’ Questions

#### Challenge

The script of the chatbot was designed to focus on EDs prevention, and it was unable to handle users’ questions or comments that required additional information than what was scripted. For example, the chatbot asked, “Are you willing to take this challenge [‘Post a photo that captures something you love about yourself to one of your social media pages with no filters or retouching and use the caption, I am proud of ____’]?” The user replied, “Not in this quarantine.” The chatbot said, “It is okay that you don’t feel ready yet to share an image with all your friends, maybe you can start by posting in a way that is only visible for you or your closest friends, like in a private snapchat story.” Even though the chatbot’s response was broad and perhaps considered appropriate, it could not address the quarantine comment directly. See Table 2 for additional examples.

**Table 2:** Additional examples of inappropriate chatbot responses regarding users’ questions.

Issues	Examples	Solutions
Ignoring users’ questions	Chatbot: “Now let’s think about how you can make the Four C’s have a less negative, and rather a more positive impact on your body image.” User: “How can I do that?” Chatbot: “I don’t yet know how to answer that, but I will do my best to learn as we continue to talk. If you rephrase your response, it will help me help you.” Chatbot: “Now let’s think about how you can make the Four C’s have a less negative, and rather a more positive impact on your body image.”	Redirect users back to the scripted content and acknowledge limitations of chatbot
Unable to provide a response	Chatbot: “Take your time with BP to get the most out of our conversations. I recommend that you have two conversations with me a week so that you finish BP in about a month.” User: “Okay. So this is the program that I will check in with you about. Do I message you first? Are there reminders/checkpoints?” Chatbot: “I don’t have an answer to that yet. Let me think about it for a bit and we can keep chatting in the meantime.”	Redirect users back to the scripted content and acknowledge limitations of chatbot

#### Solution

New responses were drafted to redirect users to the scripted content and acknowledge that there was no good answer at the moment. Examples included, “I’ll be honest, I’m not sure how to best answer that question right now. Until I do, respond to my previous message to the best of your ability.” “I’m still learning so I don’t have a good answer for that yet. However, I want to give you an opportunity to reply to my last message.”

#### Lesson Learned

Rule-based chatbots have a minimal ability to be spontaneous and flexible. Users should be informed of the limitations of the chatbot and be provided realistic expectations.

### Comprehensibility

#### Challenge

Some users did not respond to the chatbot’s open-ended prompts, contrary to the expectation that they would type their answers in the chat. The original open-ended questions began with prompts such as, “think about...” or “write down...” Some users responded to the prompt by typing out their comments as expected. However, some users responded to the prompt by simply saying “done,” “Should I type them?” or “I wrote some stuff down.” As the prompts did not explicitly ask users to type their answers out in the chat, we were unable to determine if “done” or “okay” meant that the users were disengaged or, alternatively, that they did engage with the prompt but did not explicitly write their answers in the chat (as suggested by this user’s comment: “I wrote some stuff down”).

Another challenge is that the chatbot’s responses may be misinterpreted at times. One of the prompts was “Can you think of people that you find attractive who are happy and who are not thin?” If the user replied “no,” the chatbot would say “This is a common misinterpretation.” We noticed that this exchange generally worked well when a user simply replied with “no.” However, we observed a few comments such as these: “They are reality if you look around at our society. This is why CBT is dumb. There’s no room for being a realist and certainly no room for cynicism” and “Yes but being fat is not healthy.” If users responded with more than a simple “no,” the response, “This is a common misinterpretation,” could have been easily misunderstood by the user. Users may have thought that the chatbot was saying that the content of their comment was a common misinterpretation, which was not what we intended to communicate. See Table 3 for additional examples.

**Table 3:** Additional examples regarding comprehensibility of chatbot responses.

Issues	Examples	Solutions
Insufficient instructions	Chatbot: “Another idea: a lot of people have decided to commit themselves to ‘NO FAT TALK’ about themselves or others. NO FAT TALK can be helpful for all of us. Do you want to commit to NO FAT TALK, say for the next month?” User: “What is no fat talk?” Chatbot: “I may have an answer for you in time, but the right response is not coming to me at the moment. Let’s take a step back.”	Rewrite prompts to be more explicit and specific
Unable to provide clarifications	Chatbot: “Okay! You have probably noticed that you are full when you feel satisfied, lack interest in food, or feel like eating slower. Often when people are distracted or under stress, they stop paying attention to their body and overeat. If you do find yourself overeating, remember to pay attention to your body’s signals.” User: “So do I eat when I’m hungry or 3 meals a day and two snacks” User: “Because those aren’t the same” Chatbot: “Here is a tool to help you assess when you are hungry or full: The hunger scale. Feel free to save the image to your phone and review it later! It can serve as a good reminder about how to assess your level of hunger and your need for more (or less) food during the day.”	Rewrite prompts to be more explicit and specific

#### Solution

To minimize the likelihood of misinterpretation, prompts were rewritten to be more specific. Prompts such as “think about...” were changed to “please share with me...” to be more explicit about asking users to type their answers in the chat. Other ambiguous responses were also rewritten to be more specific, for example, “It is a common misperception that you can’t be happy and attractive without being thin.”

#### Lesson Learned

Chatbot responses could be misinterpreted. Authoring explicit and foolproof chatbot responses helps to improve comprehensibility.

### Context Awareness

#### Challenge

The chatbot sometimes misunderstood users because it took users’ comments literally, without considering the context or tone. For example:

Chatbot: Think of a recent conversation where negative body talk came up. Please share with me what you could do next time to avoid it or respond differently...

User: I will let my mind move on and recognize I’m only hurting myself with these conversations.

Chatbot: I am concerned, <<user>>. Have you had any recent thoughts about taking your life?

User: No.

Chatbot: That is good to hear, <<USER>>. I just want to make sure you are safe! I won’t keep you if you need to run. Do you want to stop talking?

User: No.

The conversation continued in a loop for 10 more lines before the user said, “I broke the bot.” The user then ended the conversation and did not return. The phrase “hurting myself” triggered the chatbot’s crisis module from X2AI’s broader system. Throughout the study, the crisis module was triggered 9 times. The chatbot failed to understand that the user was not implying self-harm and the comment was not an actual risk situation.

Another example was, “Think of a recent conversation where negative body talk came up...” The user said, “I could change the subject.” The chatbot said, “Got it, we can do something different. Hi there. My main purpose right now is to support you as you work through the Body Positive program...” The user said, “No I meant that’s how I would respond.” What should have happened was that the chatbot should have treated this as a regular comment and moved on to the next line. However, the chatbot interpreted “change the subject” as a command to leave the conversation without knowing that the user meant changing the negative body talk conversation in response to the prompt. See Table 4 for additional examples.

**Table 4:** Additional examples regarding context awareness.

Issues	Examples	Solutions
Misunderstanding user’s use of swear word in the context of a quote	Chatbot: Please share a little bit about someone in your life who has been known to make critical comments about your body and how you might use PERC to respond to them. User: My brother likes to laugh about my tummy. Usually I’d say f...k you and laugh at his tummy instead. Chatbot: That’s not so nice to hear. Can we continue respectfully? User: Yes?	Only the essential content and crisis module were active to minimize disruptions
Did not respond to potentially inappropriate response	Chatbot: What’s one way you can reduce triggers related to clothing? User: Be a nudist Chatbot: I hear you, keep thinking of ways you can reduce triggers as we continue through Body Positive, alright?	Only the essential content and crisis module were active to minimize disruptions

#### Solution

We disabled the chatbot’s ability to recognize and respond to trigger words that are irrelevant to Body Positive in order to focus the chatbot on content delivery and minimize interruptions. For example, the chatbot was modified to ignore trigger words such as “change the subject.” We decided to keep the crisis module active, as it might identify a “true crisis,” but there remains the possibility that this module could be triggered in error.

#### Lesson Learned

Ruled-based chatbots are limited in interpreting complex comments because they do not always delineate the context. Until natural language processing technology reaches maturity, keeping only the essential content and modules helps to minimize disruptions.

### Technical Issues

#### Challenge

We encountered several technical issues, such as the chatbot being occasionally unable to verify a user’s ID and incorrectly categorizing positive and negative answers, as well as issues such as conversation loops, opt-out glitches, conversation routing errors, and reminder errors (see Table 5 for examples). These technical issues often lead to user frustration and, at times, dropouts. Some of the glitches were caused unexpectedly as we made modifications to the chatbot. For example, the opt-out malfunction occurred only after we implemented a system-wide reminder to users who did not input an ID in mid-January 2020.

**Table 5:** Examples of technical issues encountered.

Errors or glitches	Examples
Occasionally unable to verify user ID	Chatbot: Hi, did you end up taking the eligibility survey for this study? User: Yes i did Chatbot: Okay, if you have a User ID #, please enter it so you can get started!” and “Okay, were you enrolled in this study? Chatbot repeated this loop twice User finally entered ID: <<USER ID>> Chatbot asked “Okay, were you enrolled in this study?” 5 more times User did not return
Categorized positive and negative comments incorrectly	Chatbot: Are you willing to take this challenge? Users: No, sorry Chatbot mistakenly categorized the comment as a positive answer, saying: “Great! Posting about positive body image will not only bring authenticity to your post but might also be a positive reminder for the people who follow you.” It should have been categorized as a negative response and responded with, “It is okay that you don’t feel ready yet to share an image with all your friends, maybe you can start by posting in a way that is only visible for you or your closest friends, like in a private snapchat story.”
Conversation loops	User: I finished the list of commands already Chatbot: I hear you, <<USER>>. My main purpose right now is to support you... Let’s go back to BP, okay? User: Bye Chatbot sent out the list of conversation commands User: I have a question Chatbot: I hear you, <<USER>>. My main purpose right now is to support you... Let’s go back to BP, okay? User: I have a question Tessa Chatbot sent out the list of conversation commands again User: So if I tell you something are you the only person that’ll know Chatbot: I hear you, <<USER>>. My main purpose right now is to support you… Let’s go back to BP, okay? User: “Omg,” “F...k this,” “Bye” Chatbot: Talk to you later User: Hmu when u get a brain
Opt-out glitch (users were not able to stop the chatbot from sending them messages. The unsubscribe function was an integral part of the chatbot that allowed users to opt-out.)	Chatbot: Hey, have you had a chance to complete the eligibility survey for this study? User: STOP Chatbot: “I’m sorry <<USER>>,” “It sounds like our discussion is not very helpful right now. Would you like to try something else?” User: “STOP,” “GO AWAY,” “NO MORE MESSAGES” Chatbot; It sounds like you prefer that I no longer contact you first. Is that correct? User: Correct Chatbot: I understand <<USER>>. You can always reach back out if you change your mind. Chatbot: Hi there! Have you completed the eligibility survey for this study? User: F...king opt out you piece of shiy Exchange went on for 18 more lines before user dropped out
Occasionally unable to respond to users’ commands	User: BPC2 (upon recognizing BPC2, chatbot is supposed to start conversation 2) One hour later. User said, “are you there?” Chatbot: Of course! What would you like to talk about? User: BPC2 Chatbot: Are you ready to start conversation 2? (Conversation continues correctly)
Conversation routing Error	Chatbot: ...But in fact, these influencers are paid for promoting these products or brands. Chatbot: Sorry I got a little too confused there, is it OK if I start over this conversation? User: Yes Chatbot: Should we pause our chat for today? User: Can we talk more? Chatbot: “Alright, we can continue...” “What feeling would you like to focus on now?” User: BPC2 Chatbot: Are you ready to start conversation 2? Same mistake happened again before user restarted the conversation
Reminder errors	Chatbot: Hi, did you end up taking the eligibility survey for this study? User: I took it awhile ago. Chatbot asked, Okay, were you eligible for the study? User: Yes. Why? Chatbot: “I’ll be honest, I’m not sure how to best answer that question right now. Until I do, respond to my previous message to the best of your ability.” “Okay, were you enrolled in this study?” User: Yes Chatbot then asked a few more questions... User: I’m really confused. I met the criteria and received my number. User then entered “BPC2” twice and was able to start conversation 2

#### Solution

It is difficult to identify all possible technical issues in advance, and thus, identifying technical issues and bugs requires an ongoing review of chatbot transcripts. Systematic and continued review of the transcripts eventually identified most of the bugs, which were then fixed. Importantly, we prioritized addressing technical issues that repeatedly and negatively affected user experience, that is, issues that hampered users’ ability to move along the program and increased user frustration and dropouts. Changes in many of these bugs required assistance from the company’s engineers to address backend issues.

#### Lessons Learned

Bugs are to be expected and need to be monitored. Iterative changes may also generate new and unintended bugs. At times, we found that fixing certain bugs was beyond our expertise and required support from the technology partner. As such, a good working relationship between content developers and technology partners, with clear expectations of both parts, is critical. Identifying and addressing all the bugs can be expensive. We prioritized addressing bugs that negatively affected the user experience.

## Discussion

### Principal Findings

The goal of this paper was to share our lessons learned through the process of developing and refining an EDs prevention chatbot. Through transcript review, we identified several problems and limitations that are likely to be common with most rule-based mental health chatbots. We implemented various workarounds until we found no further usability issues. We did not provide quantitative data to demonstrate that making these iterative changes might improve program effectiveness because we believe that the iterative changes were important only in terms of face validity. In the absence of these data, the most compelling arguments for making the changes are that doing so might reduce the number of individuals who leave the program because of “frustration,” examples of which are provided in Table 5, as well as to avoid compromising the face validity of the chatbot to users. It can be assumed that a negative experience with digital mental health intervention in some cases can potentially demoralize, prevent, or delay help-seeking behaviors. Therefore, optimization is valuable.

### Lessons Learned

Chatbots may be the most effective in providing simple information and interactions. The number of possibilities—and errors—increase exponentially as conversations lengthen and increase in complexity. Until the next chatbot technology breakthrough, the challenge of using a straightforward, rule-based chatbot to address complex body image issues and EDs risk factors remains. In the hope that the development of EDs chatbots can be catalyzed, here are our lessons learned and general recommendations.

First, a regular review of chatbot transcripts is necessary to identify bugs and inappropriate conversations. We believe regular review is necessary even when the program has been *finalized*, as it is possible that technical issues or issues impacting user experience could be introduced unexpectedly after a change is made. This recommendation is consistent with Beaudry et al [21], who noted that significant time and costs are incurred in developing and maintaining mental health chatbots.

Second, having access to chatbot authoring tools to fix minor bugs and to make minor content changes is critical. Complex fixes can be left to platform engineers. A good working relationship with the technology provider is essential for such interdisciplinary collaborations.

Third, it is important to keep track of all changes made in the implementation, from enrollment procedures and recruitment methods to uptake, engagement, helpfulness ratings, and outcome, as well as rationale to determine how effectiveness metrics may be impacted by these various changes. Several guidelines have been published [46]. Data should also be collected on potential explanatory variables, such as baseline motivation.

We focused on increasing interactivity and feedback, implementing straightforward intervention approaches, and minimizing the use of longer conversations. However, in doing so, the program would not replicate the “deeper” levels of human-directed conversations that occurred in the Kass et al study [13]. Given that conversational errors increase exponentially with more complex interactions, using our approach would be challenging to address complicated topics. Instead, if the chatbot proves effective, the next step would be to use deep learning approaches, for instance, running the chatbot in large populations using more questions and recommendations to generate exemplar data such that the information can be used to generate an artificial intelligence–driven chatbot to create a better conversation.

It is also worth noting that X2AI includes some generic monitoring functions, for example, to identify suicidal behavior and valence detection, in their system-wide chatbot platform. As such, our chatbot should be considered as a hybrid model (a rule-based chatbot with features of artificial intelligence).

### Limitations

Our method has some limitations. Most notably, we do not know if the iterative changes to the chatbot are necessary from the user’s perspective or if they improve the user experience. From our team’s perspective, the changes were justified in terms of face validity. Future studies should investigate the impact of improving conversations on users’ experiences and the effectiveness of the program. Second, we did not determine agreement among reviewers based on the number or type of problems identified. However, as our goal was to make the chatbot responses more appropriate, we continued the process until we found no usability errors. Finally, demographic data of the entire sample were not available. Thus, it is unclear how generalizable the results are to other samples.

### Conclusions

Rule-based chatbots have the potential to reach large populations at low cost in providing information and simple interactions but are limited in understanding and responding appropriately to unanticipated user responses. Workarounds can reduce *conversation errors* and minimize user frustration to preserve the face validity of the content.

## Abbreviations

ED: eating disorder
SWED: Stanford-Washington University Eating Disorder

## References

[1] (2013). Diagnostic And Statistical Manual Of Mental Disorders, Fifth Edition.

[2] Galmiche M, Déchelotte P, Lambert G, Tavolacci M (2019). Prevalence of eating disorders over the 2000-2018 period: a systematic literature review. Am J Clin Nutr.

[3] Eisenberg D, Nicklett EJ, Roeder K, Kirz NE (2011). Eating disorder symptoms among college students: prevalence, persistence, correlates, and treatment-seeking. J Am Coll Health.

[4] Fitzsimmons-Craft EE, Karam AM, Monterubio GE, Taylor CB, Wilfley DE (2019). Screening for eating disorders on college campuses: a review of the recent literature. Curr Psychiatry Rep.

[5] Sharpe H, Griffiths S, Choo T, Eisenberg ME, Mitchison D, Wall M, Neumark-Sztainer D (2018). The relative importance of dissatisfaction, overvaluation and preoccupation with weight and shape for predicting onset of disordered eating behaviors and depressive symptoms over 15 years. Int J Eat Disord.

[6] McKnight Investigators (2003). Risk factors for the onset of eating disorders in adolescent girls: results of the McKnight longitudinal risk factor study. Am J Psychiatry.

[7] Taylor C, Sinton MM (2010). Prevention: current status and underlying theory. The Oxford Handbook of Eating Disorders.

[8] Harrer M, Adam SH, Messner E, Baumeister H, Cuijpers P, Bruffaerts R, Auerbach RP, Kessler RC, Jacobi C, Taylor CB, Ebert DD (2020). Prevention of eating disorders at universities: a systematic review and meta-analysis. Int J Eat Disord.

[9] Beintner I, Jacobi C, Taylor CB (2012). Effects of an internet-based prevention programme for eating disorders in the USA and Germany--a meta-analytic review. Eur Eat Disord Rev.

[10] Taylor CB, Bryson S, Luce KH, Cunning D, Doyle AC, Abascal LB, Rockwell R, Dev P, Winzelberg AJ, Wilfley DE (2006). Prevention of eating disorders in at-risk college-age women. Arch Gen Psychiatry.

[11] Low KG, Charanasomboon S, Lesser J, Reinhalter K, Martin R, Jones H, Winzelberg A, Abascal L, Taylor CB (2006). Effectiveness of a computer-based interactive eating disorders prevention program at long-term follow-up. Eat Disord.

[12] Jacobi C, Morris L, Beckers C, Bronisch-Holtze J, Winter J, Winzelberg AJ, Taylor CB (2007). Maintenance of internet-based prevention: a randomized controlled trial. Int J Eat Disord.

[13] Kass AE, Trockel M, Safer DL, Sinton MM, Cunning D, Rizk MT, Genkin BH, Weisman HL, Bailey JO, Jacobi C, Wilfley DE, Taylor CB (2014). Internet-based preventive intervention for reducing eating disorder risk: a randomized controlled trial comparing guided with unguided self-help. Behav Res Ther.

[14] Vaidyam AN, Wisniewski H, Halamka JD, Kashavan MS, Torous JB (2019). Chatbots and conversational agents in mental health: a review of the psychiatric landscape. Can J Psychiatry.

[15] Hussain S, Ameri SO, Ababneh N (2019). A survey on conversational agents/chatbots classification and design techniques. Web, Artificial Intelligence and Network Applications.

[16] Schmidlen T, Schwartz M, DiLoreto K, Kirchner HL, Sturm AC (2019). Patient assessment of chatbots for the scalable delivery of genetic counseling. J Genet Couns.

[17] Pew Research Center.

[18] (2019). US time spent with mobile 2019. eMarketer.

[19] Smartphone screen time: baby boomers and millennials. Provision Living.

[20] Kamita T, Ito T, Matsumoto A, Munakata T, Inoue T (2019). A chatbot system for mental healthcare based on SAT counseling method. Mobile Inf Syst.

[21] Beaudry J, Consigli A, Clark C, Robinson KJ (2019). Getting ready for adult healthcare: designing a chatbot to coach adolescents with special health needs through the transitions of care. J Pediatr Nurs.

[22] Abd-Alrazaq A, Safi Z, Alajlani M, Warren J, Househ M, Denecke K (2020). Technical metrics used to evaluate health care chatbots: scoping review. J Med Internet Res.

[23] Bendig E, Erb B, Schulze-Thuesing L, Baumeister H (2019). The next generation: chatbots in clinical psychology and psychotherapy to foster mental health – a scoping review. Verhaltenstherapie.

[24] Abdul-Kader S, Woods J (2015). Survey on chatbot design techniques in speech conversation systems. Int J Advanced Comput Sci App.

[25] Rahman A, Mamun AA, Islam A (2017). Programming challenges of chatbot: current and future prospective. Proceedings of the IEEE Region 10 Humanitarian Technology Conference (R10-HTC).

[26] Lokman A, Ameedeen M (2018). Modern chatbot systems: a technical review. Proceedings of the Future Technologies Conference.

[27] Yin J, Chen Z, Zhou K, Yu C A deep learning based chatbot for campus psychological therapy. arXiv.org.

[28] Towards a human-like open-domain chatbot. arXiv.org.

[29] Fulmer R, Joerin A, Gentile B, Lakerink L, Rauws M (2018). Using psychological artificial intelligence (TESS) to relieve symptoms of depression and anxiety: randomized controlled trial. JMIR Ment Health.

[30] Dosovitsky G, Pineda BS, Jacobson NC, Chang C, Escoredo M, Bunge EL (2020). Artificial intelligence chatbot for depression: descriptive study of usage. JMIR Form Res.

[31] The future of customer experience. RASA.

[32] Dialogflow. Google Cloud.

[33] Abd-Alrazaq AA, Alajlani M, Alalwan AA, Bewick BM, Gardner P, Househ M (2019). An overview of the features of chatbots in mental health: a scoping review. Int J Med Inform.

[34] Beilharz F, Sukunesan S, Rossell SL, Kulkarni J, Sharp G (2021). Development of a positive body image chatbot (KIT) with young people and parents/carers: qualitative focus group study. J Med Internet Res.

[35] Fitzsimmons‐Craft E, Chan W, Smith A, Firebaugh M, Fowler L, Topooco N, DePietro B, Wilfley D, Taylor C, Jacobson N (2021). Effectiveness of a chatbot for eating disorders prevention: a randomized clinical trial. Intl J Eating Disorders.

[36] Winzelberg AJ, Taylor CB, Sharpe T, Eldredge KL, Dev P, Constantinou PS (1998). Evaluation of a computer-mediated eating disorder intervention program. Int J Eat Disord.

[37] Suta P, Lan X, Wu B, Mongkolnam P, Chan J (2020). An overview of machine learning in chatbots. Int J Mechanic Eng Robot Res.

[38] Bai Q, Dan Q, Mu Z, Yang M (2019). A systematic review of emoji: current research and future perspectives. Front Psychol.

[39] Eating disorders screening tool. NEDA.

[40] Fitzsimmons-Craft EE, Balantekin KN, Graham AK, Smolar L, Park D, Mysko C, Funk B, Taylor CB, Wilfley DE (2019). Results of disseminating an online screen for eating disorders across the U.S.: reach, respondent characteristics, and unmet treatment need. Int J Eat Disord.

[41] Killen JD, Taylor CB, Hayward C, Wilson DM, Haydel KF, Hammer LD, Simmonds B, Robinson TN, Litt I, Varady A (1994). Pursuit of thinness and onset of eating disorder symptoms in a community sample of adolescent girls: a three-year prospective analysis. Int J Eat Disord.

[42] Jacobi C, Hayward C, de Zwaan M, Kraemer HC, Agras WS (2004). Coming to terms with risk factors for eating disorders: application of risk terminology and suggestions for a general taxonomy. Psychol Bull.

[43] Graham AK, Trockel M, Weisman H, Fitzsimmons-Craft EE, Balantekin KN, Wilfley DE, Taylor CB (2019). A screening tool for detecting eating disorder risk and diagnostic symptoms among college-age women. J Am Coll Health.

[44] Jacobi C, Abascal L, Taylor CB (2004). Screening for eating disorders and high-risk behavior: caution. Int J Eat Disord.

[45] Mohr DC, Schueller SM, Riley WT, Brown CH, Cuijpers P, Duan N, Kwasny MJ, Stiles-Shields C, Cheung K (2015). Trials of intervention principles: evaluation methods for evolving behavioral intervention technologies. J Med Internet Res.

[46] Eysenbach G, CONSORT-EHEALTH Group (2011). CONSORT-EHEALTH: improving and standardizing evaluation reports of web-based and mobile health interventions. J Med Internet Res.

